# Sartor Resartus

**Published:** 1888-06

**Authors:** M. W. Vandenburg

**Affiliations:** Fort Edward, N. Y.


### SARTOR RESARTUS.

In the May number of this journal, Dr. McLaren takes me to task for "some very surprising statements," and I fully agree with him as regards the typo's pranks. Let me correct those first. Top of page 78, second line, was written, one point, not "our point;" the large bulk of the sick, not "rich" (unfortunately). The Doctor is right; "brain" as printed was written pain; and the biggest somersault is still to come, in the third line from the bottom, where *vis medicatrix naturæ* is made to read, "his medicative nature," which is, to say the least, a rather free translation.

Dr. McLaren gives three very pretty cases of homœopathic treatment of gall stones. But we cannot help asking, Were all of your cases, Doctor, as easily managed as these three? Was relief as prompt and cure as complete? There are cases, no doubt, where Morphia fails to ease the pain. I have heard of such, but have not chanced to meet them. There are cases to my certain knowledge that have been relieved by Morphia, and have had no return of the disease for over six years. I can vouch for at least half a dozen such. Now, does this prove that Morphia cured them? Hardly, I think, though some of our friends of the other persuasion might have the hardihood to assert that this was the case.

Only one word farther regarding the use of Morphia. The writer is not a Morphiaphobist. He uses it both by hypodermic and by the mouth. One case is living who took hypodermics of at least a grain each, sometimes of two grains each, every four hours for fifteen days. This was a case of traumatic tetanus of the worst description, and in the course of the attack involved every muscle, from crown to sole. Now, my dear Doctor, do not run to the extreme from this hint. The use of Morphia is very exceptional. Probably once a week would be a high average. But I am not afraid to use it when it is adjuvant to homœopathic remedies, nor am I, like a good ex-President of the American Institute, whom I overheard talking of his personal case, say that he was ashamed to own that he used Morphia on himself.

I know of no reason why a homœopathic physician should not use adjuvants, but I will not quarrel with those who will not use them. Will the Doctor kindly tell us whether he has ever made any failures in *immediately relieving* calculi?

As to Hahnemann's explanation of, and his remarks on, mesmerism and "its efficacy," the less said the better. Otherwise, a great majority of the profession might be adjudged "madmen." M. W. VANDENBURG, A. M., M. D.

FORT EDWARD, N. Y.

